# The Treatment of Disabled Soldiers

**Published:** 1916-02-19

**Authors:** 


### THE TREATMENT OF DISABLED SOLDIERS.

THE more prominent medical and surgical relations of the war have naturally been those concerned with acute contingencies: that is, with epidemic or endemic diseases, and with the immediate surgery of wounds. Another problem is gradually attracting attention, and it is one of large importance. We refer to the position created by the existence in considerable numbers of soldiers who on discharge from hospital remain in a more or less disabled condition as a result of their injuries. Many of these are permanently unfitted for further military service, and, unless suitable help is afforded, they will also be permanently unfitted for the occupations of civil life. Such a result is a serious matter alike for the individual and for the community, and this both in a moral and in an economic sense. There is abundant ground for stating that many of these men can by suitable treatment be restored to a fair degree of activity, and with this possibility in view a clear responsibility rests on the medical profession to see that the appropriate measures are instituted and are made available for those who need them. These measures, to describe them in brief, consist of such mechanical agencies as baths, systematic exercises, ordered movements, massage, and electricity. Means for the application of such agencies do exist in some degree, but the demand is already in excess of the supply, and the disproportion is likely rather to grow than to diminish.

In these circumstances it is important that every encouragement be offered to the committee appointed by the Balneological Section of the Royal Society of Medicine. Steps have been already taken to inform the Medical Department of the Army of the opportunities which actually exist for both hydrological and mechanical forms of treatment, and at several centres numbers of soldiers have been received and treated. More recently it has deputed one of its members to study a special combination of methods which has been practised at an institution in Paris. That there is need for insistence we do not doubt. Rarely does the official world welcome information taking origin outside its sacred fold, and in the present emergency it must be admitted that this world has acute claims on its attention. Even on the professional side some agitation may be necessary. The freeing of stiff joints and the restoration of atrophied muscles make a much less dramatic appeal than the brilliant operation or the hunt for a new bacillus. We have nothing but applause for these latter activities, but the former also deserve attention, and their utilitarian value under existing conditions can hardly be overstated. The committee, we think, might usefully press for the appointment of a special officer in the Medical Department of the Army and charged with the duty of providing for all who need it the kind of treatment which is here discussed. Amidst more acute cases, the care of the disabled men is in danger of being overlooked unless someone is specifically charged with a responsibility that covers the whole position. To meet the national responsibility in this respect the medical profession must insist on the provision of adequate opportunities and equipment.

